# A novel homozygous missense variant p.D339N in the *PKLR* gene correlates with pyruvate kinase deficiency in a Pakistani family: a case report

**DOI:** 10.1186/s13256-022-03292-z

**Published:** 2022-02-16

**Authors:** Atta Ur Rehman, Abdur Rashid, Zubair Hussain, Khadim Shah

**Affiliations:** 1 Department of Biomedical Sciences, Pak-Austria Fachhochschule: Institute of Applied Sciences and Technology, Khanpur Road, Mang, Haripur, Pakistan; 2 Department of Higher Education Archives and Libraries Peshawar, Government of Khyber Pakhtunkhwa, Peshawar, Pakistan; 3 Department of Biotechnology, COMSATS University Islamabad, Abbottabad Campus, Abbottabad, Pakistan

**Keywords:** Consanguinity, Pakistan, *PKLR* variant, Homozygous, PK deficiency, Case report

## Abstract

**Background:**

Pyruvate kinase deficiency is an exceptionally rare autosomal recessive Mendelian disorder caused by bi-allelic pathogenic variants in the *PKLR* gene. It is mainly characterized by chronic nonspherocytic hemolytic anemia though other symptoms such as splenomegaly, hepatomegaly, pallor, fatigue, iron overload, shortness of breath, hyperbilirubinemia, and gallstones might also prevail.

**Case presentation:**

We present here a novel genetic defect in the *PKLR* gene that correlates with pyruvate kinase deficiency phenotype in a consanguineous family from North-Western Pakistan. The family included three affected individuals who were all born to consanguineous parents. The proband, a 13-year-old female of Pashtun ethnicity, showed chronic nonautoimmune hemolytic anemia since birth, extremely low hemoglobin (7.6 g/dL) and pyruvate kinase (12.4 U/g Hb) levels, splenomegaly, and hepatomegaly. Bone marrow aspirate showed a markedly decreased myeloid to erythroid ratio and hypercellular marrow particles due to hyperplasia of the erythroid elements. Molecular characterization of the proband’s genomic DNA uncovered a likely pathogenic homozygous missense variant p.[D339N] in exon 7 of the *PKLR* gene. In-depth *in silico* analysis and familial cosegregation implies p.[D339N] as the likely cause of pyruvate kinase deficiency in this family. Further *in vitro* or *in vivo* studies are required to validate the impact of p.[D339N] on protein structure and/or stability, and to determine its role in the disease pathophysiology.

**Conclusions:**

In summary, these findings suggest a novel genetic defect in the *PKLR* gene as a likely cause of pyruvate kinase deficiency, thus further expanding the mutational landscape of this rare Mendelian disorder.

**Supplementary Information:**

The online version contains supplementary material available at https://doi.org/10.1186/s13256-022-03292-z.

## Background

Pyruvate kinase deficiency (PKD) is recognized mainly by chronic nonspherocytic hemolytic anemia (CNSHA), though other symptoms such as splenomegaly, hepatomegaly, pallor, fatigue, iron overload, shortness of breath, hyperbilirubinemia, and gallstones may also prevail, thus showing that PKD presents considerable clinical variability across patients [1]. The symptoms may range from few or no clinical indications to more severe and life-threatening anemia, especially in childhood [1]. PKD affect people of all races, however, the prevalence rate is not uniform across countries. It appears to affect about 51 per one million people of Western ancestry [2, 3]. The condition arises due to genetic defect in the *PKLR* gene (pyruvate kinase, liver and red cell isoform), and is inherited in an autosomal recessive pattern [4]. Located on chromosome 1q21, *PKLR* gene encodes a glycolytic enzyme called pyruvate kinase (PK), critical for glucose metabolism (glycolysis), adenosine triphosphate (ATP) production, and the energy balance of cells [5].

Thus far, over 300 pathogenic or likely pathogenic variants, predominantly missense substitutions, have been reported in the *PKLR* gene [6]. A correct diagnosis of PKD requires the identification of pathological changes in the *PKLR* gene, along with subsequent confirmation of their impact on PK enzymatic activity. This is important due to the fact that all sequence variants in the *PKLR* gene are not necessarily causative, as observed in some patients with homozygous or compound heterozygous changes in the *PKLR* gene but having normal PK activity [7, 8]. Instead, some *PKLR* variants have been found to be beneficial by providing protection against malarial infections, both in mice and humans [9]. Thus, the *PKLR* gene has been under strong selection pressure in countries where malaria is endemic, for instance, Pakistan and Sub-Saharan Africa [10, 11].

Currently, no approved therapeutic options are available for correcting PKD [12]. Existing management of PK deficiency mostly include supportive treatments such as transfusion of red blood cells (RBCs), iron chelation therapy, and/or splenectomy [5]. Nonetheless, these supportive treatments have numerous inherent risks, notably pulmonary hypertension, thrombosis, iron loading, osteopenia, gallstones, and extramedullary hematopoiesis [13–15]. To the best of our knowledge, no previous study documenting a molecular cause of PK deficiency in Pakistan is available thus far. In this investigation, we report a novel molecular defect in the *PKLR* gene likely causing PK deficiency in a consanguineous family from North-Western Pakistan.

## Methods

This study was initiated following a formal authorization (Approval No. F.NO:185/HU/Zool/2021/182) from the Institutional Review Board of Hazara University, Mansehra, and written approval of informed consent by the guardian of the family. Clinical data were extracted from the available medical records while pedigree was drawn electronically using Pedigree Chart Designer software (CeGaT GmbH, Tübingen, Germany). Saliva samples were obtained by the Oragene DNA collection kit (Genotek, Ottawa, Canada) from six participating individuals of the family. The participants included the proband, proband’s mother, paternal grandparents, paternal uncle, and his wife. DNA was extracted from the saliva samples following ethanol precipitation protocol as mentioned in the prepIT.L2P manual. Quantitative and qualitative assessment of DNA was made using a spectrophotometer and 1% agarose gel, respectively. To PCR amplify coding regions and exon–intron boundaries of the *PKLR* gene (NM_000298.6; NP_000289.1), a total of eight exon-specific primer pairs were designed using Primer3web (version. 4.1) [16]. Primers sequences and PCR cycles are shown in Additional file 1: Table S1 and S2, respectively. Briefly, the PCR comprised of the following steps: Initial denaturation of template DNA at 95 °C for 5 minutes, followed by 35 PCR cycles each at 95 °C for 30 seconds (denaturation step), 57 °C for 30 seconds (primer annealing step), 72 °C for 30 seconds (elongation step), and a final elongation step at 72 °C for 5 minutes. PCR products were purified using ExoSAP-IT reagent (Catalog # 78200, Thermo Fisher Scientific, USA) prior to Sanger sequencing of the products using a commercial facility.

## Case presentation

### Clinical data and family information

**Fig. 1:**
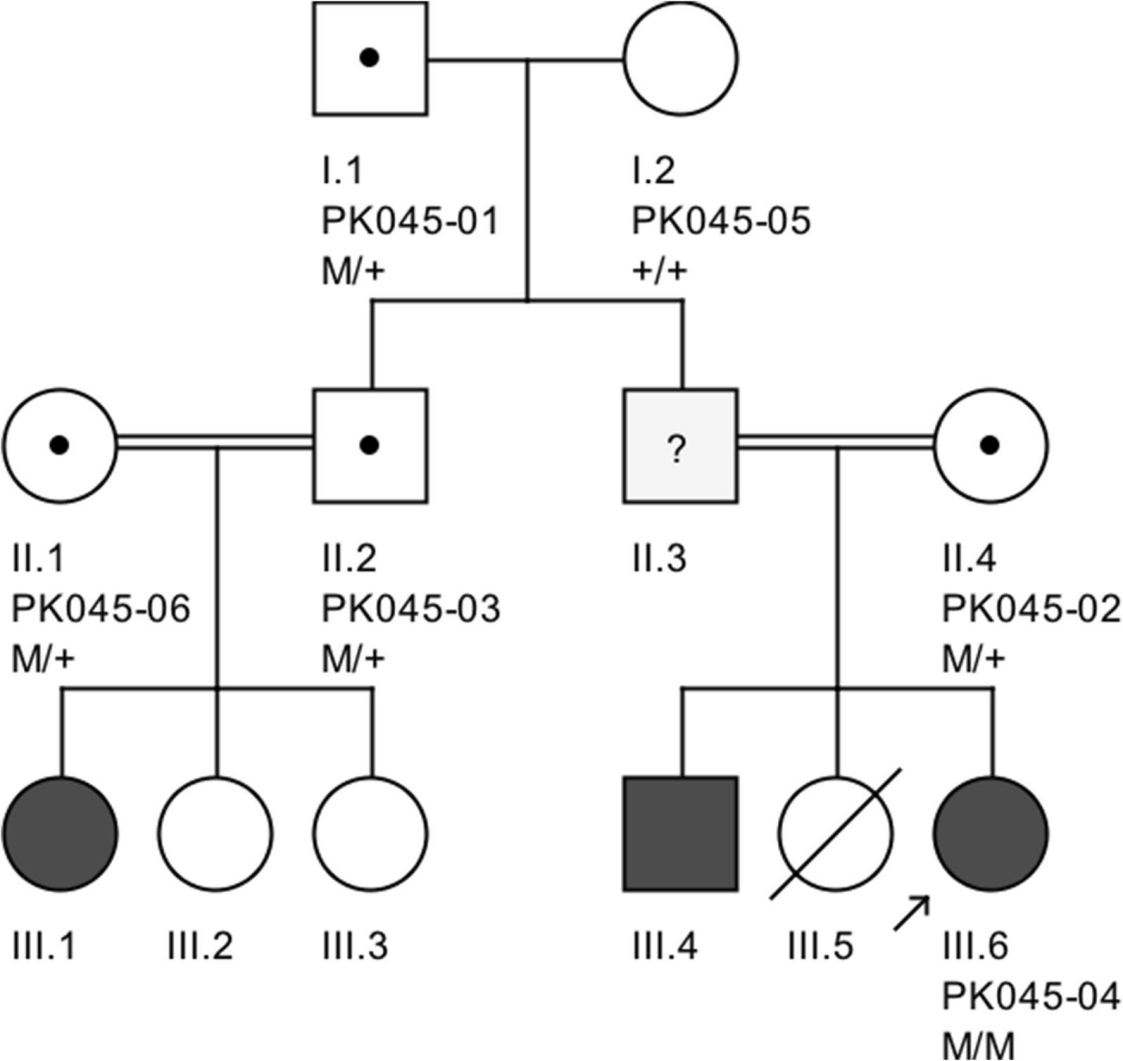
Pedigree of a Pakistani family segregating *PKLR* mutation. Filled symbols indicate patients while blank symbols represent healthy individuals. Symbols carrying a central dot shows obligate carriers. M = Mutation; + = Wild-type allele. Proband in the pedigree is indicated by a small arrow

We characterized, both clinically and genetically, a consanguineous Pakistani family suffering from pyruvate kinase deficiency (PKD). The family belonged to a Pashtun ethnic group living in the Peshawar municipality, and consisted of three patients; the proband (III.6), her brother (III.4), and one first cousin (III.1), all born to consanguineous parents (Fig. 1). However, genetic testing was performed on the proband only. The proband is currently a 13-year-old female who was born full term to a consanguineous couple. At clinical examination, the proband experienced chronic, most likely congenital nonautoimmune hemolytic anemia at birth, and thus was recommended for transfusion. Transfusion was started regularly since she was 22 days old, with a frequency of once a month to once every 3 months. Along with transfusion, an oral supplementation of folic acid 1 mg, daily was recommended for 30 days to stabilize the patient’s hemoglobin level. The proband had an axillary temperature of 36.1 °C, peripheral pulse rate of 114 beats per minute, respiratory rate of 24 breaths per minute, systolic blood pressure of 94 mm Hg, diastolic blood pressure of 500 mm Hg, oxygen saturation of 99%, height of 94.3 cm, weight of 13.4 kg, body mass index of 15.1 kg/m^2^, and body surface area of 0.59 m^2^. The proband’s height and weight remained at the third percentile though her both parents were relatively tall, likely indicating a lack of expected normal physiological development in childhood. The proband’s blood group type was AB-negative. Immunization was up to date, and no known allergies were revealed upon clinical investigation. Echocardiogram (echo) was unremarkable. Examination of the musculoskeletal, neurologic, lymphatic, and integumentary systems revealed no adverse outcomes. Abdominal examination revealed hepatomegaly (palpable, 2.6 cm below the right costal margin, smooth, not tender), splenomegaly (palpable, 2 cm below left costal margin, smooth edged, not tender; spleen size 8.8 cm). Bone marrow aspirate showed a markedly decreased myeloid to erythroid (M/E) ratio, and marked hypercellular marrow particles due to hyperplasia of the erythroid elements with normal maturation. However, myeloid maturation was normal and the number of megakaryocytes were also within the normal range, thus excluding evidence of red cell aplasia, myelodysplastic syndrome, or congenital dyserythropoietic anemia (CDA). Screening for paroxysmal nocturnal hemoglobinuria (PNH) was also negative. Measurements of blood hemoglobin (Hb) and pyruvate kinase (PK) levels were extremely low at 7.6 g/dL and 12.4 U/g Hb, respectively (Table 1). Based on the clinical findings, a final diagnosis of pyruvate kinase deficiency (PKD) was confirmed in the proband.

**Table 1:** Clinical and genetic data of a Pakistani family with pyruvate kinase deficiency.

Demographic and clinical information		Genetic findings	
Age	13 years	Basic variant information	
Sex	Female	Chromosome location	1q22
Residence	Peshawar	Genomic position	155264127
Ethnicity/language	Pashtun/Pashto	Gene symbol	PKLR
ABO, Rh blood type	AB, negative	Gene name	Pyruvate kinase L/R
Hemoglobin (Hb)	7.6 gm/dL	Ensembl Gene ID	ENSG00000143627
Pyruvate kinase (PK)	12.4 U/g Hb	OMIM ID	609712
Spleen	Palpable, splenomegaly (spleen size 8.8 cm)	Transcript ID	NM_000298.6
Liver	Palpable, hepatomegaly	Protein ID	NP_000289.1
Temperature (Axillary)	36.1 °C	Exon number	7
Peripheral pulse rate	114	cDNA change	c.1015G > A
Respiratory rate	24 br/minute	Protein change	p.Asp339Asn
Systolic blood pressure	94 mm Hg	Variant type	SNV
Diastolic blood pressure	50 mm Hg	Variant status	Novel
Oxygen saturation	0.99	RS ID	rs747097960
Height (third percentile)	94.3 cm	*In silico* analysis	
Weight (third percentile)	13.4 kg	gnomAD (All) MAF	0.00001592
Body surface area (BSA)	0.59 m^2^	gnomAD (South Asian) MAF	0.0001307
Body mass index (BMI)	15.1 kg/m^2^	gnomAD homozygotes	0
Folic acid	1 mg, PO, daily, 30 days	ACMG classification	Likely pathogenic
Transfusion started	22 days after birth	ClinVar/HGMD	N/A
Transfusion frequency	Once a month to once every 3 months	DEOGEN2	Damaging
Allergies	No known allergies	LRT	Deleterious
Bone marrow biopsy	Marked erythroid hyperplasia with marked reticulocytosis	MutPred	Pathogenic
Musculoskeletal	Normal strength	Mutation assessor	Highly damaging
Lymphatics	No lymphadenopathy	MutationTaster	Disease causing
Integumentary	No rash	PROVEAN	Damaging
Neurologic	Alert	PrimateAI	Damaging
Sodium level	137 mmol/L	REVEL	Pathogenic
Potassium level	4.1 mmol/L	SIFT	Damaging
Chloride Level	109 mmol/L	Polyphen-2	Probably damaging
CO_2_	22 mmol/L	FATHMM-MKL	Damaging
AGAP	6 mmol/L	DANN	0.9993
Miscellaneous	Lack of expected normal physiological development in childhood	CADD PHRED score (GRCh37-v1.6)	29.5
Final diagnosis	Pyruvate kinase deficiency	Evolutionary conservation score (PhyloP100way)	7.564

### Genetic findings

**Fig. 2:**
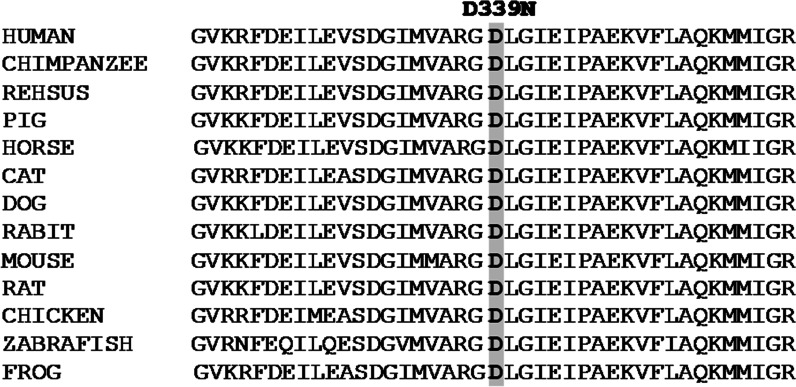
Species-wise conservation of the altered PKLR residue [p.D339N]. Multiple sequence alignment of PK orthologs showing that wild-type aspartic acid (highlighted bold font in gray) is highly conserved across vertebrate species

**Fig. 3:**
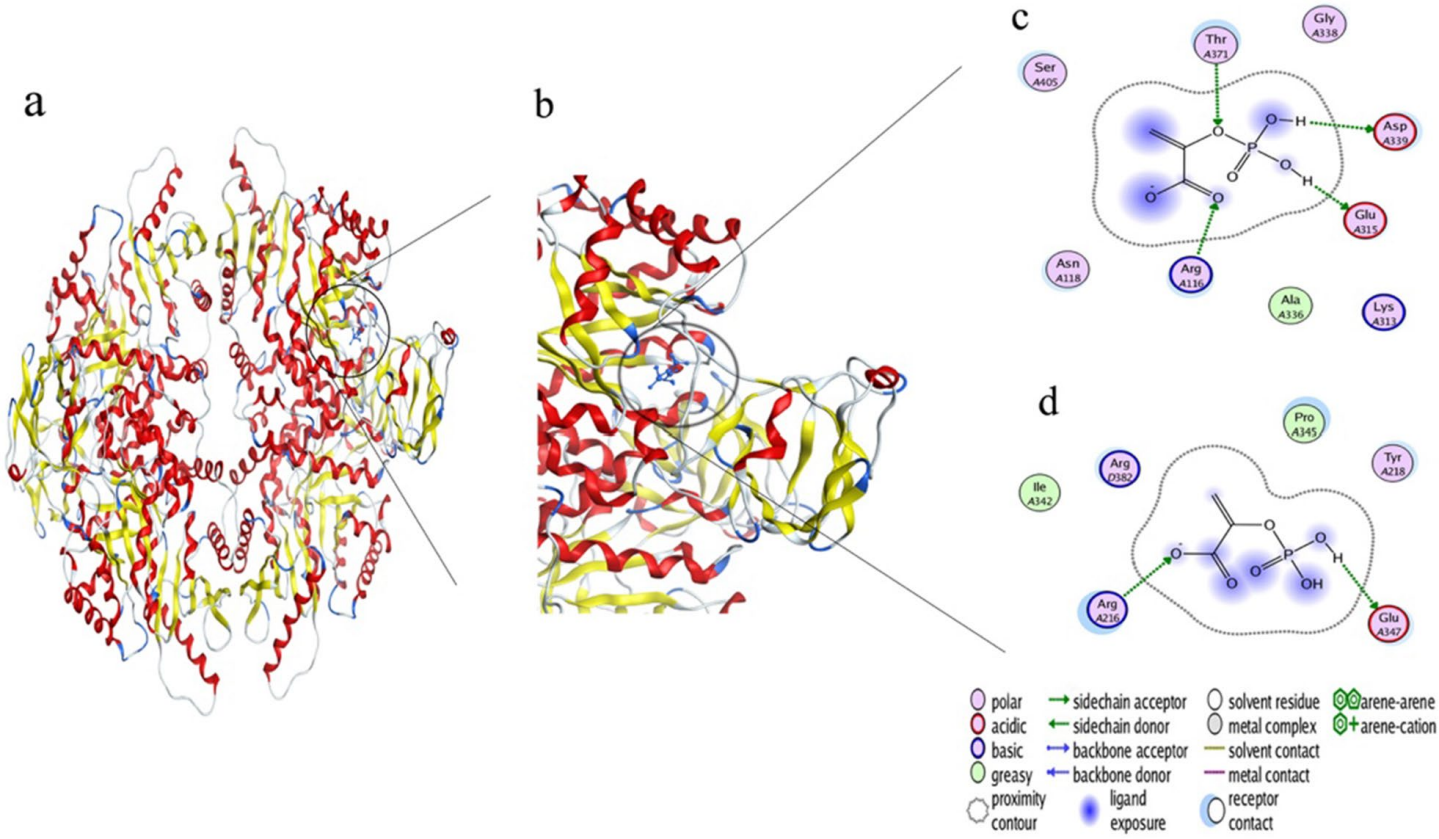
Three dimensional (3D) modeling of the PKLR protein structure. **a** Wild PK tetramer with the ligand and **b** closer view of the phosphoenolpyruvate site in the complex. **c** showing the interactions of wild PK with phosphoenolpyruvate through Arg116, Glu316, and Asp339, and **d** mutant PK and phosphoenolpyruvate interactions through Arg216 and Glu347

Sanger sequencing revealed a likely pathogenic homozygous missense variant (c.1015G > A) in exon 7 of the *PKLR* gene, resulting in a single amino acid substitution p.[D339N] in the PK protein. The variant p.[D339N] cosegregated with PKD phenotype in the studied family (Fig. 1). For instance, the variant was present in a homozygous state in the proband while none of the clinically unaffected family members carried the variant in a homozygous state. Of the five unaffected family members who participated in this study, four were heterozygous for the variant, while one was homozygous for the wild-type allele. To the best of our knowledge, the variant c.1015G > A has never been associated with PKD phenotype nor previously reported in the ClinVar or the Human Gene Mutation Database (HGMD). The variant was present in the gnomAD database with extremely low minor allele frequency (MAF 0.00001592); however, the allele was not present in a homozygous state. Existing *in silico* tools and the American College of Medical Genetics and Genomics (ACMG) classified the variant as “Likely pathogenic” (Table 1). Multiple sequence alignment of the PK orthologs showed highest conservation of Asp339 residue across vertebrate species (Fig. 2), thus reflecting the importance of Asp339 residue for PK activity. To find out the effect of this mutation on the protein’s 3D structure, we modeled wild and mutant protein structures using an online method [17]. Similarly, we performed docking using MOE software to evaluate protein–ligand interaction [18]. These computational analyses revealed that wild-type PK interact with phosphoenolpyruvate through three residues including Arg116, Glu316, and Asp339. However, the mutant protein (p.[D339N]) lost its normal interactions with phosphoenolpyruvate and developed unusual interactions through Arg216 and Glu347 (Fig. 3). Altogether, our findings suggest that p.D339N mutation possibly reduces or abolishes PK enzymatic activity leading to PK deficiency in the affected people.

## Discussion and conclusions

PKD is a rare autosomal recessive Mendelian disorder caused by mutations in the *PKLR* gene [4, 6]. Clinically, PKD appears with diverse symptoms ranging from few or no clinical indications to more severe and life-threatening anemia such as CNSHA, especially in childhood [1]. So far, more than 300 sequence variants in the *PKLR* gene have been associated with PKD in different ethnic groups across the globe [6]. Most of these sequence variants are missense substitutions affecting residues critical to the structure and/or function of the protein, followed by frameshift and splicing mutations and non-sense; promoter variants and large indels are rare. Recently, compound heterozygous variants with deep intronic mutations have been reported as a cause of PK deficiency (19). Clinical data and *in vitro* analysis showed that more severe phenotypes are commonly coupled with disruptive sequence variants (stop codon, frameshift, splicing, and large deletions) and with missense variants directly involved in active site or protein stability (8). In Pakistan, which has a high proportion of consanguineous marriages and several patients with PKD, to the best of our knowledge, a sequence variant of the *PKLR* gene has never been reported in PKD patients. This reflects the limitation of molecular studies on PKD patients in the Pakistani population.

This study correlates a novel genetic defect in the PKLR gene with PK deficiency in a consanguineous Pashtun family of North-Western Pakistan. The proband’s hallmark symptoms included CNSHA appearing since birth, extremely low hemoglobin (7.6 g/dL) and pyruvate kinase (12.4 U/g Hb) levels, splenomegaly, and hepatomegaly. Bone marrow aspirate showed a markedly decreased myeloid to erythroid (M/E) ratio, and hypercellular marrow particles due to hyperplasia of the erythroid elements. Molecular characterization of the proband’s genomic DNA revealed a likely pathogenic homozygous missense variant p.[D339N] in exon 7 of the *PKLR* gene. Furthermore, based on the docking results, we speculate that the loss of normal protein–ligand interactions due to p.[D339N] results in poor or no dephosphorylation of phosphoenolpyruvate by the mutant protein, resulting in an energy production defect in glycolysis.

Historically, *PKLR* gene has been under strong selection pressure in malaria-endemic countries, notably, Pakistan and Sub-Saharan Africa [10, 11], though hundreds of *PKLR* mutations are known to cause PK deficiency in humans [9]. Furthermore, the rate of PK deficiency well correlates with the prevalence of consanguinity in countries such as Turkey [19], Iraq [20], Saudi Arabia [21], Iran [22], and the Amish population [23] due to bi-allelic expression of recessive mutations. In Pakistan, an estimated 3.1% neonates with hemolytic anemia have clinically confirmed PK deficiency [24]. However, to the best of our knowledge, no further genetic investigation has been performed to delineate the molecular cause of PKD.

In conclusion, our findings suggest a novel genetic defect in the *PKLR* gene as a likely cause of PK deficiency in a consanguineous Pakistani family, and thus possibly constitute the first-ever *PKLR* mutation reported from Pakistan. In addition to expanding the mutational spectrum of this rare monogenic disorder, our study warrants further *in vitro* or *in vivo* studies to validate the functional impact of p.[D339N] on the protein structure and/or stability.

## Supplementary Information

**Additional file 1: Table S1**. Primers used for PCR amplification of all exons of *PKLR* gene (NM_000298.6). **Table S2**. PCR conditions/cycles used in this study.

## Data Availability

The datasets used and/or analyzed during the current study are available from the corresponding author on reasonable request.

## Abbreviations

ACMG: The American College of Medical Genetics and Genomics
CNSHA: Chronic nonspherocytic hemolytic anemia
DNA: Deoxyribonucleic acid
ExAC: Exome aggregation consortium
LOVD: Leiden open variation database
PCR: Polymerase chain reaction
PKD: Pyruvate kinase deficiency
HGMD: Human gene mutation database
gnomAD: Genome aggregation database
