# Supplementary material for: A novel homozygous missense variant p.D339N in the PKLR gene correlates with pyruvate kinase deficiency in a Pakistani family: a case report
Source: J Med Case Rep. 2022 Feb 16;16:66. doi: 10.1186/s13256-022-03292-z (PMC8848962; doi:10.1186/s13256-022-03292-z)
Supplement: Supplementary file 1 — Additional file 1: Table S1. Primers used for PCR amplification of all exons of PKLR gene (NM_000298.6). Table S2. PCR conditions/cycles used in this study. [file 13256_2022_3292_MOESM1_ESM.docx]

**Legends:**

**Table S1.** Primers used for PCR amplification of all exons of *PKLR* gene (NM_000298.6).

**Table S2.** PCR conditions/cycles used in this study.

**Table S1.**

| **Exon #** | **Forward Primer** | **Reverse Primer** | **Product Size (bp)** |
| --- | --- | --- | --- |
| Exon 1 | caggagatgagggcagagag | tttaacacacgggaggctct | 426 |
| Exon 2 | gaagggaaggaacagagggt | ccagggcccaaaatcttgtc | 432 |
| Exon 3-4 | gggaaggggagtctgtgatc | agatgtgagttctgagcccc | 599 |
| Exon 5 | gtggagagctttgcaggttc | atagtccagcccaaacccag | 596 |
| Exon 6-7 | cctctctgctgcaactgtg | cactacttgggaggctgagg | 800 |
| Exon 8-9 | tgagagtgtgggtgtcagag | attgctgcctctcctcttgt | 635 |
| Exon 10 | caccaaggcccagagaagta | tgtgtggctatgctgatgga | 509 |
| Exon 11 | tcactgcaacctctacctcc | gaggacttaaaggtggggct | 469 |

**Table S2.**

| **PCR Step** | **Temperature** | **Time** |  |
| --- | --- | --- | --- |
| Initial Denaturation | 95 °C | 5 Minutes |  |
| Denaturation | 95 °C | 30 Seconds | **35-cycles** |
| Primer Annealing | 57 °C | 30 Seconds |  |
| Elongation | 72 °C | 45 Seconds |  |
| Final Elongation | 72 °C | 5 Minutes |  |
| PCR Stop | 10 °C | -- |  |
